# Evaluation of user experiences, perceptions and attitudes towards faecal immunochemical testing (FIT) for risk-stratified colonoscopy in people with Lynch syndrome

**DOI:** 10.1136/bmjgast-2025-001751

**Published:** 2025-05-19

**Authors:** Anne Lincoln, Jo Waller, Sally Benton, FIT for Lynch Study Collaborators, Richard Martin, Peter Sasieni, Kevin J Monahan

**Affiliations:** 1 King’s College London, London, England, UK; 2 QMUL, London, England, UK; 3 Royal Surrey County Hospital NHS Foundation Trust, Guildford, Surrey, UK; 4 Centre for Familial Intestinal Cancer, St Marks Hospital, London, London, UK; 5 Surgery and Cancer, Imperial College London, London, UK

**Keywords:** HNPCC SYNDROME, INHERITED CANCERS, CANCER SYNDROMES, CANCER PREVENTION, STOOL MARKERS

## Abstract

**Objective:**

This study evaluates the experiences, perceptions, and attitudes of people with Lynch syndrome (LS) towards faecal immunochemical testing (FIT) as an adjunct to colonoscopy for colorectal cancer surveillance.

**Methods:**

A mixed-methods design included two cohorts: an emergency clinical service during the COVID-19 pandemic and a longitudinal research initiative. Paper-based surveys assessed user experiences and attitudes using Likert scales and open-ended questions. Quantitative data were analysed for response patterns, while thematic analysis explored qualitative feedback.

**Results:**

Among 85% of participants who rated FIT positively, 90% were confident in using the test correctly. Women reported higher anxiety (7.8%) compared with men (2.0%, p=0.045). Thematic analysis revealed gratitude for FIT’s convenience during healthcare disruptions but emphasised concerns about its accuracy.

**Conclusions:**

FIT is widely perceived as an acceptable supplemental tool among surveyed people with LS, who appreciate its potential to reduce intervals between colonoscopies without compromising surveillance quality. Ongoing patient engagement is crucial to addressing concerns about FIT’s reliability. Future research should evaluate long-term outcomes and explore diverse population perspectives to guide FIT implementation in LS surveillance.

**WHAT IS ALREADY KNOWN ON THIS TOPIC**

**WHAT THIS STUDY ADDS**

- This study shows that FIT is generally well-accepted by people with LS, with most finding it easy to use. Significant differences in user experiences were observed, with women reporting higher levels of anxiety related to CRC surveillance, while older respondents expressed greater confidence in FIT.

**HOW THIS STUDY MIGHT AFFECT RESEARCH, PRACTICE, OR POLICY**

- Our findings indicate that FIT is generally well-received as a potential adjunct to colonoscopy in LS surveillance, but further research is needed to evaluate its long-term impact and optimise patient communication strategies.

## Introduction

 Lynch syndrome (LS) is the most common inherited cause of colorectal cancer (CRC), accounting for approximately 3% of all CRC cases.^1^ People with LS have a significantly increased lifetime risk of developing CRC and other malignancies due to inherited mutations in the DNA mismatch repair genes *MLH1*, *MSH2*, *MSH6* and *PMS2*, and deletions in the *EPCAM* gene which regulate *MSH2* expression.^2 3^

Colonoscopy remains the primary surveillance tool for CRC in people with LS, but it is often burdensome due to the need for frequent procedures, associated anxiety, and delays in accessing care. A recent study highlights these challenges, revealing that colonoscopy surveillance in people with LS is frequently delayed, increasing the risk of advanced CRC and exacerbating patient burden.^4^ The strain that frequent colonoscopies place on healthcare systems and patients emphasises the need for alternative or supplementary approaches, such as faecal immunochemical testing (FIT), which could mitigate these delays and alleviate the procedural burden.

Given these challenges, FIT has been explored as a potential adjunct to colonoscopy in patient populations with an elevated lifetime risk of CRC^5^,^7^ with promising findings to suggest it may be used at specific intervals between colonoscopies to detect advanced neoplasia (AN) at an early stage. Nevertheless, FIT’s diagnostic performance in surveillance depends on the faecal haemoglobin (f-Hb) threshold (measured by micrograms of Hb per gram of faeces (µg/g)) used to define a positive result. Though lower thresholds may enhance early detection of AN, it may also lead to higher false-positive rates. Conversely, higher thresholds may improve specificity but risk missing significant lesions.^8^

However, there is little published data about the attitudes of people with LS towards different screening modalities, including FIT. As FIT is routinely used in population-based screening programmes,^9 10^ attitudes towards this test, as well as faecal occult blood testing, are well-documented in average-risk screening and in high-risk populations, such as those with symptoms,^11^,^14^ as well as in polypectomy surveillance^15 16^ and familial cohorts.^17^ However, the potential utility of FIT in people with LS has only recently been explored and, as such, the attitudes and preferences regarding FIT in LS patient populations have not been previously evaluated. Understanding these viewpoints is essential to effectively assess the potential utility of FIT in this high-risk population.

This study investigates the acceptability of FIT, and attitudes and perceptions towards its use as a surveillance tool within an LS population, based on two distinct projects: an emergency clinical service^18^ and the FIT for Lynch Study^19^ (Projects 1 and 2). Project 1 was implemented across England during the COVID-19 pandemic to prioritise timely colonoscopy for people with LS through the use of FIT for risk stratification, while Project 2 is a prospective, longitudinal research initiative in England and Scotland exploring the long-term feasibility and acceptability of FIT as a surveillance tool in a postpandemic setting. Combining these cohorts provides a comprehensive understanding of FIT’s utility across emergency and routine care, revealing shared attitudes despite differing contexts.

## Methods

### Study design

This cross-sectional survey study including both precoded and free text items, evaluated FIT user experience, attitudes, and perceptions of FIT as a surveillance tool among people with LS. The research integrated quantitative and qualitative analysis to assess participant experiences and attitudes. This study adheres to the Consensus-Based Checklist for Reporting of Survey Studies^20^ for comprehensive and transparent reporting (see online supplemental figure 1).

### Participants

Participants were drawn from two distinct initiatives: an emergency clinical service in England using FIT for risk-stratified colonoscopy prioritisation during the COVID-19 pandemic (Project 1),^18^ and a prospective research study, ‘FIT for Lynch’ (Project 2).^19^ In Project 1, FIT results influenced colonoscopy timing, prioritising urgent cases. While FIT did not guide routine clinical management in Project 2, an additional colonoscopy was recommended if f-Hb concentration met or exceeded a predefined threshold of 6 µg of Hb per gram of faeces (µg/g) during the intervening study years between scheduled surveillance colonoscopies.

Cohorts from both projects were selected based on predefined eligibility criteria, which included an LS diagnosis confirmed by germline testing, irrespective of prior CRC history, and English proficiency. For Project 1 eligibility required a planned surveillance colonoscopy between 1 March 2020 and 31 March 2021. In Project 2, the age criteria were 25–73 years for individuals with pathogenic variants in *MLH1*, *MSH2*, and *EPCAM*, and 35–73 years for *PMS2* or *MSH6* mutation carriers, as based on the UK CRC surveillance guidelines for LS. Exceptions were made at the principal investigator’s discretion for individuals aged 18–35 with prior colonoscopy, particularly those with significant family or personal history of CRC who were advised to start surveillance earlier than current guidelines stipulate.^21^ Participants in the Project 1 cohort completed their surveys amidst significant healthcare disruptions. Conversely, recruitment for Project 2 took place between late 2021 and mid-2023, a period marked by the easing of national lockdown restrictions and the resumption of clinical services following the widespread availability of COVID-19 vaccinations in the UK.

### Recruitment

Across both projects, individuals with confirmed LS pathogenic variants were identified by authorised clinical and research staff from participating National Health Service (NHS) Trusts. Recruitment strategies included screening clinical records, direct clinician referrals, and outreach through NHS genetic services. Eligible participants were contacted by their local clinical teams and provided with study information before enrolment.

While Project 1 and Project 2 were distinct initiatives, some NHS sites participated in both, meaning certain individuals may have been contacted for both studies. However, recruitment processes remained independent, with separate screening and consent processes for each project. Participation in Project 1 did not guarantee enrolment in Project 2, and some individuals who took part in Project 1 may have declined Project 2. These factors were considered to maintain the integrity and independence of each study. Further details on recruitment, eligibility criteria, and participant selection strategies are extensively documented in our previously published studies.^18 19^

^18 19^

### Data management

A sole assigned data manager used standardised data entry templates, ensuring consistency across all responses. Random accuracy checks were performed to verify data integrity before analysis

### Procedures

#### Project 1

Participants received a FIT pack by mail, which included a FIT kit (OC-Sensor, Eiken Chemicals, Japan), a biohazard bag, a prepaid return envelope, and a double-sided survey (see online supplemental figure 2). Survey completion was encouraged alongside the return of the FIT kit. FIT instructions provided by the kit supplier (Mast) were modified with minor revisions to suit study requirements.

#### Project 2

Participants received a baseline study mailing containing a FIT kit (OC-Sensor, Eiken Chemicals, Japan), informed consent form, and a double-sided baseline survey. The survey structure mirrored that of Project 1 where participants completed the user experience and FIT attitudes survey questionnaire alongside other measures (see online supplemental figure 3). In addition to FIT instructions, Project 2 participants received a patient information sheet and a study informational poster, which outlined key study details and reinforced that FIT was intended as a supplemental tool, rather than a replacement for colonoscopic surveillance. These materials are detailed in Lincoln *et al*.^19^

### Measures

#### Participant survey design

A paper-based survey was developed using theoretical frameworks previously used to examine novel, self-sampling medical interventions in patient populations.^22 23^ Questions were then refined to assess overall experience using FIT, as well as attitudes specific to the use of FIT in people with LS. The survey included two questionnaires: the first assessed overall user experience (‘user experience questionnaire’), while the second assessed attitudes and perceptions of FIT (‘FIT attitudes questionnaire’).

### User experience

This questionnaire contained six items with response options on 4-point Likert scales. Overall experience using FIT was rated from 1 (poor) to 4 (excellent). The remaining items assessing unpleasantness, embarrassment, anxiety, and confidence in both FIT usage and accuracy were coded numerically and in ascending order, with negative responses assigned a score of 1, and positive responses assigned a score of 4. Items were reverse scored as needed. Missing responses were excluded from statistical analyses (see online supplemental figure 2).

### FIT attitudes

This questionnaire consisted of six items with response options on a 5-point Likert scale, ranging from strongly disagree (1) to strongly agree (5), evaluating participants’ perceptions of key aspects of FIT use. These included its reassurance value, preference for home completion, feelings of care from the NHS, trust in FIT results, and perceived improvement in surveillance with annual FIT (see online supplemental figure 2). Higher scores reflected more positive attitudes, with reverse coding applied where necessary. Items were selected to address concerns and preferences commonly identified in studies on novel screening tools, particularly focusing on people with LS’ trust in FIT as a reliable and convenient supplement to colonoscopic surveillance.^24^

In addition to these six items, Project 1 included a single-item, item asking, ‘How important is it to you to have your biennial colonoscopy?’ Responses were coded from ‘Not Important’ = 1 to ‘Very Important’ = 4.

### Open-ended questions

Two open-ended questions were included for additional feedback in Project 1 only. Participants provided their thoughts on the effectiveness of FIT during the COVID-19 pandemic and any other comments or feedback.

### Additional survey content for project 2

While the surveys for both projects shared a core set of questions focused on user experience and attitudes towards FIT, the Project 2 survey also included questions related to participants’ current medication usage and ‘notable GI-related symptoms’ (see online supplemental figure 3). These questions were included to provide broader clinical context for the ongoing longitudinal study. However, these data points were not used in the analyses or write-up of this mixed-methods paper.

### Data analysis

All statistical analyses were conducted using IBM SPSS Statistics (V.29.0). Thematic analysis was carried out on written responses from the open-ended sections of the Project 1 respondent surveys using NVivo 12 Pro analysis application software. Graphical figures were generated using Microsoft Excel. Analyses included:

- Descriptive and inferential statistics.
- Associations between survey responses, age group, and sex.
- Thematic analysis of open-ended responses.

The descriptive analysis included proportions (reported as percentages) for demographic categoric variables (sex, age range, LS genotype). The sample size for both Project 1 and Project 2 was determined by the number of returned and completed surveys. While no formal power calculation was performed for Project 1 as it was exploratory and aimed to describe attitudes towards FIT in this population, power calculations were conducted to arrive at a predetermined sample size for Project 2, as detailed by Lincoln *et al*.^19^ Missing items from both questionnaires and the open-ended section in Project 1 were coded as ‘9999’ and were excluded case-wise from statistical analyses.

### Distribution of responses

The distribution of responses from the user experiences questionnaire was graphically displayed using bar graphs (Microsoft Excel).

### Testing associations between item outcomes, sex, age, and LS genotype

Pearson χ² was used to test for associations between each experience and attitude item and age (<55 vs ≥55 and <52 vs ≥52, based on respondents’ median age for Projects 1 and 2, respectively), sex, and LS genotype. LS genotypes were categorised as *MLH1*/*MSH2*/*EPCAM* (higher-risk genotypes) versus *MSH6*/*PMS2* (lower-risk genotypes), based on CRC risk and known pathogenicity. For Project 1, genotype information was missing for nine participants. While these individuals had a confirmed LS diagnosis and met all other eligibility criteria, they were excluded from the χ² analysis involving LS genotypes due to the lack of specific genotype data. Significance was prespecified at 5%.

In cases where>20% of cells demonstrated expected frequencies<5, Fisher’s exact test was applied, with a significance threshold of 5%. To conduct 2×2 χ² analyses on this data set, response outcomes were dichotomised. For the user experiences questionnaire, the first two response outcomes (eg, excellent and good; very/fairly) were combined, as were the last two (eg, fair and poor; mildly/not). For FIT attitudes items, responses were grouped into strongly disagree/disagree/indifferent versus agree/strongly agree.

### Identifying response patterns through qualitative thematic analysis

Responses were coded inductively to identify recurring themes—such as concerns about the effectiveness of FIT, perceptions of its utility during the COVID-19 pandemic, and general feedback on the experience of using the test. These themes were grouped into three broader categories—‘negative’, ‘neutral’, and ‘positive’—to classify participants’ attitudes. For example, scepticism or concern about FIT was categorised as ‘negative’, satisfaction or appreciation as ‘positive’, and ambivalence or lack of strong opinion as ‘neutral’. This structured approach provided insights into common patterns across responses, enhancing understanding of participants’ perceptions and experiences with FIT. One-worded, vague, or non-applicable responses (ie, ‘thanks’ or ‘all good’) were not considered for analysis.

## Results

### Project 1

Of 339 individuals who returned a FIT kit and were deemed eligible for Project 1 participation, 281 also returned a survey. One survey was returned blank, resulting in 280 (82.6%) usable responses. Most respondents were female (64.3%), with a mean age of 52.1 years (SD=14.3). The most common LS pathogenic variant was *MSH2* (32.1%), followed by *MLH1* (31.4%) (see table 1).

**Table 1:** Combined demographics table (Project 1 vs Project 2)

Characteristic	Project 1 (emergency clinical service)	Project 2 (FIT for Lynch study)
Total respondents (n)	280	393
Mean age (SD**)**	52.1 (14.3)	51.9 (12.5)
Age group		
≤25	7 (2.5)	3 (0.8)
26–35	40 (14.3)	43 (10.9)
36–45	49 (17.5)	79 (20.1)
46–55	65 (23.2)	101 (25.7)
56–65	57 (20.4)	108 (27.5)
66–75	53 (18.9)	55 (14.0)
75	9 (3.2)	4 (1.0)
Sex		
Male	100 (35.7)	148 (37.7)
Female	180 (64.3)	245 (62.3)
LS genotype		
*MLH1*	88 (31.4)	119 (30.3)
*MSH2*	90 (32.1)	135 (34.4)
*MSH6*	67 (23.9)	82 (20.9)
*PMS2*	25 (8.9)	55 (14.0)
*EPCAM*	1 (0.4)	2 (0.5)
Missing/unknown	8 (2.9)	–

Data are presented as counts and percentages (n (%)) unless otherwise stated.

FIT, faecal immunochemical testing; LS, Lynch syndrome.

### FIT user experience

The User Experience Questionnaire revealed that 88.5% of respondents rated their overall experience with FIT as ‘Excellent’ or ‘Good’. A small minority expressed feelings of unpleasantness (1.4%), embarrassment (2.9%), or anxiety (5.7%) while using FIT. Confidence in the correct use of the FIT kit was reported by 86.7% of respondents, and 73.9% were confident in the accuracy of FIT to detect advanced colorectal neoplasia (ACN). The only significant demographic association identified was between gender and feelings of anxiety, with female respondents reporting higher levels of anxiety (7.8%) compared with male respondents (2.0%) (p=0.045) (see table 2). No significant associations were found between user experience and age. LS genotype analysis showed that participants with lower-risk *MSH6*/*PMS2* variants were marginally more confident in the accuracy of FIT compared with those with higher-risk *MLH1*/*MSH2*/*EPCAM* variants, (p=0.053; table 2), though this difference did not reach statistical significance.

**Table 2:** Project 1 (emergency clinical service) assessment of user experience-based item associations and respondent demographics

How would you rate your overall experience of the FIT kit?					
Variables	N	Excellent/good*, n (%)	Fair/poor*, n (%)	χ²	P value
All participants	278 (98.9)	246 (88.5)	32 (11.5)		
Sex				0.056	0.813
Male	99 (35.6)	87 (87.9)	12 (12.1)		
Female	179 (64.4)	159 (88.8)	20 (11.2)		
Age (years)				NA†	0.828
≥55	127 (45.7)	114 (89.8)	13 (10.2)		
<55	151 (54.0)	132 (87.4)	19 (12.6)		
LS genotype‡				0.141	0.708
*MLH1/MSH2/EPCAM*	177 (65.8)	155 (87.6)	22 (12.4)		
*MSH6/PMS2*	92 (34.2)	82 (89.1)	10 (10.9)		
**Did you find the FIT kit unpleasant?**					
**Variables**	**N**	Not/mildly unpleasant*, n (%)	Fairly/very unpleasant*, n (%)	**χ²**	**P value**
All participants	279 (99.6)	275 (98.6)	4 (1.4)		
Sex				NA†	0.620
Male	100 (35.8)	98 (98.0)	2 (2.0)		
Female	179 (64.2)	177 (98.9)	2 (1.1)		
Age (years)				NA†	1.000
≥55	127 (45.5)	125 (98.4)	2 (1.6)		
<55	152 (54.5)	150 (98.7)	2 (1.3)		
LS genotype‡				NA†	0.607
*MLH1/MSH2/EPCAM*	178 (65.9)	2 (1.2)	176 (98.9)		
*MSH6/PMS2*	92 (34.1)	2 (2.2)	90 (97.8)		
**Did you feel embarrassed using the FIT kit?**					
**Variables**	**N**	Not/mildly embarrassed*, n (%)	Fairly/very embarrassed*, n (%)	**χ²**	**P value**
All participants	280 (100)	272 (97.1)	8 (2.9)		
Sex				NA†	0.054
Male	100 (35.7)	100 (100.0)	0 (0.0)		
Female	180 (64.3)	172 (95.6)	8 (4.4)		
Age (years)				NA†	1.000
≥55	128 (45.7)	124 (96.9)	4 (3.1)		
<55	152 (54.3)	148 (97.4)	4 (2.7)		
LS genotype‡				NA†	0.720
*MLH1/MSH2/EPCAM*	179 (66.1)	173 (96.7)	6 (3.3)		
*MSH6/PMS2*	92 (33.9)	90 (97.8)	2 (2.2)		
**Did you feel anxious while using the FIT kit?**					
**Variables**	**N**	Not/slightlyanxious*, n (%)	Fairly/veryanxious*, n (%)	**χ²**	**P value**
All participants	279 (99.6)	263 (94.3)	16 (5.7)		
Sex				4.022	**0.045**
Male	100 (35.8)	98 (98.0)	2 (2.0)		
Female	179 (64.2)	165 (92.2)	**14** (**7.8**)		
Age (years)				0.137	0.711
≥55	127 (45.5)	119 (93.7)	8 (6.3)		
<55	152 (45.5)	144 (94.7)	8 (5.3)		
LS genotype‡				0.060	0.806
*MLH1/MSH2/EPCAM*	178 (65.9)	167 (93.8)	11 (6.2)		
*MSH6/PMS2*	92 (34.1)	87 (94.6)	5 (5.4)		
**How confident are you that you used the FIT kit correctly?**					
**Variables**	**N**	Very/fairlyconfident*, n (%)	Not very/not at all confident*, n (%)	**χ²**	**P value**
All participants	278 (98.9)	241 (86.7)	37 (13.3)		
Sex				0.188	0.664
Male	99 (35.6)	87 (87.9)	12 (12.1)		
Female	179 (64.4)	154 (86.0)	25 (14.0)		
Age (years)				2.044	0.153
≥55	128 (46.0)	115 (89.8)	13 (10.2)		
<55	150 (54.0)	126 (84.0)	24 (16.0)		
LS genotype‡				3.017	0.082
*MLH1/MSH2/EPCAM*	177 (65.8)	148 (83.6)	29 (16.4)		
*MSH6/PMS2*	92 (34.2)	84 (91.3)	8 (8.7)		
**How confident are you that the result you will receive for the FIT kit will be accurate in detecting any potential precancer or cancer?**					
**Variables**	**N**	Very/fairlyconfident*, n (%)	Not very/not at all confident*, n (%)	**χ²**	**P value**
All participants	272 (97.1)	201 (73.9)	71 (26.1)		
Sex				0.597	0.440
Male	97 (35.7)	69 (71.1)	28 (28.9)		
Female	175 (64.3)	132 (75.4)	43 (24.6)		
Age (years)				3.372	0.066
≥55	125 (46.0)	99 (79.2)	26 (20.8)		
<55	147 (54.0)	102 (69.4)	45 (30.6)		
LS genotype‡				3.736	0.053
*MLH1/MSH2/EPCAM*	172 (65.4)	121 (70.3)	51 (29.7)		
*MSH6/PMS2*	91 (34.6)	74 (81.3)	17 (18.7)		

Boldface indicates statistically significant values at p < 0.05.

* The first two responses and the last two responses within the 4-point Likert scale of this questionnaire were combined for dichotomisation purposes as required for 2×2 χ² analysis.

† Fisher’s Exact Test was utilised in this instance as the findings were not deemed suitable for interpretation by Pearson Chi-Square (more than 20% of the expected frequencies had a value of <5.)

‡ LS genotypes were combined in a 2x2 fashion for chi-square analysis based on CRC risk and known pathogenicity^21^

FIT, faecal immunochemical testing; LS, Lynch syndrome; NA, not applicable.

### FIT attitudes

The FIT Attitudes Questionnaire indicated that most respondents felt reassured by the availability of FIT during the COVID-19 pandemic (74.3%), with 81.8% appreciating the option to complete the FIT kit at home. Additionally, 60.0% trusted that FIT results would inform the timing of their colonoscopy, and 81.1% believed that adding an annual FIT to biennial colonoscopy would improve their LS surveillance (table 3).

**Table 3:** Project 1 (emergency clinical service) FIT attitudes

FIT attitudes items	Strongly disagree N (%)	Disagree N (%)	Indifferent N (%)	Agreen (%)	Strongly agreen (%)	No response/N/An (%)
I have been anxious about my routine colonoscopy being cancelled or postponed due to COVID-19	10 (3.6)	15 (5.4)	30 (10.7)	100 (35.7)	125 (44.6)	0 (0.0)
Having the option of using the FIT kit is reassuring	5 (1.8)	8 (2.9)	12 (4.3)	90 (32.1)	140 (50.0)	5 (1.8)
I like the idea of doing the kit in the comfort of my own home	2 (0.7)	4 (1.4)	10 (3.6)	95 (33.9)	155 (55.4)	14 (5.0)
The NHS makes me feel cared for by offering me this FIT kit while I wait for a colonoscopy	3 (1.1)	6 (2.1)	18 (6.4)	110 (39.3)	135 (48.2)	8 (2.9)
I trust that the result of the FIT test can be used to decide when I need a colonoscopy	12 (4.3)	20 (7.1)	50 (17.9)	150 (53.6)	100 (35.7)	8 (2.9)
Doing an FIT kit every year, as well as having a colonoscopy every 2 years, would improve my Lynch syndrome surveillance	4 (1.4)	5 (1.8)	15 (5.4)	120 (42.9)	145 (51.8)	11 (3.9)

Data are presented as counts and percentages (n (%)) unless otherwise stated.

* Fisher’s Exact Test was used in this instance as the findings were not deemed suitable for interpretation by Pearson χ² (more than 20% of the expected frequencies had a value of <5.).

† LS genotypes were combined in a 2×2 fashion for χ² analysis based on CRC risk and known pathogenicity.

CRC, colorectal cancer; FIT, faecal immunochemical testing; N/A, not applicable; NHS, National Health Service.

### Thematic analysis of open-ended responses

Of the 280 respondents, 83 (29.6%) provided comments in the open-ended survey sections (see online supplemental figure 2). After excluding one-worded, vague, or non-applicable responses (eg, ‘thanks’ or ‘all good’), 54 comments (65.1%) were analysed. A near-even distribution of 25 (46.3%) ‘negative’ and 23 (42.6%) ‘positive’ responses was observed, with 6 (11.1%) categorised as ‘neutral’. Although negative responses slightly outnumbered positive ones, some responses received dual coding. Themes from negative responses included misunderstandings about FIT’s intended use, concerns about its accuracy, and anxiety regarding the lack of transparent results. Positive responses generally expressed gratitude towards the NHS for providing FIT during a time of limited healthcare services and confidence in its potential as an adjunct to colonoscopy (see onlinesupplemental figures 4^6^).

### Project 2

Overall, 393 (94.2%) of 417 participants who returned a baseline FIT kit as part of Project 2 also returned a survey. Women comprised 62.3% of respondents, with a mean age of 51.9 years (SD=12.5). The most common LS pathogenic variant was *MSH2* (34.4%), followed by *MLH1* (30.3%) (table 1).

### FIT user experience

The User Experience Questionnaire results showed that 92.4% of respondents rated their overall experience with FIT as ‘Excellent’ or ‘Good’. A small proportion of respondents reported unpleasantness (3.6%), embarrassment (0.5%), or anxiety (0.8%) while using FIT. A significant majority (96.4%) reported confidence in using the FIT kit correctly, and 88.7% were confident in FIT’s accuracy for detecting ACN (see table 4).

**Table 4:** Project 2 (FIT for Lynch Study) assessment of user experience-based Item associations and respondent demographics

How would you rate your overall experience of the FIT kit?					
**Variables**	**n**	**Excellent/good** * **, n (%)**	**Fair/poor** * **, n (%)**	**χ²**	**P value**
All participants	393 (100)	363 (92.4)	30 (7.6)		
Sex				1.122	0.289
Male	148 (37.7)	134 (90.5)	14 (9.5)		
Female	245 (62.3)	229 (93.5)	16 (6.5)		
Age (years)				1.215	0.270
≥52	211 (53.7)	192 (91.0)	19 (9.0)		
<52	182 (46.3)	171 (94.0)	11 (6.0)		
LS genotype‡				3.279	0.070
*MLH1/MSH2/EPCAM*	256 (65.1)	241 (94.1)	15 (5.9)		
*MSH6/PMS2*	137 (34.9)	122 (89.1)	15 (10.9)		
**Did you find the FIT kit unpleasant?**					
**Variables**	**n**	**Not/mildly unpleasant** * **, n (%)**	**Fairly/very unpleasant** * **, n (%)**	**χ²**	**P value**
All participants	393 (100)	379 (96.4)	14 (3.6)		
Sex				0.23	0.878
Male	148 (37.7)	143 (96.6)	5 (3.4)		
Female	245 (62.3)	236 (96.3)	9 (3.7)		
Age (years)				0.070	0.792
≥52	211 (53.7)	203 (96.2)	8 (3.8)		
<52	182 (46.3)	176 (96.7)	6 (3.3)		
LS genotype‡				0.005	0.946
*MLH1/MSH2/EPCAM*	256 (65.1)	247 (96.5)	9 (3.5)		
*MSH6/PMS2*	137 (34.9)	132 (96.4)	5 (3.6)		
**Did you feel embarrassed using the FIT kit?**					
**Variables**	**n**	Not/mildly embarrassed*, n (%)	Fairly/very embarrassed*, n (%)	**χ²**	**P value**
All participants	392 (99.7)	390 (99.5)	2 (0.5)		
Sex				NA†	0.142
Male	148 (37.8)	146 (98.6)	2 (1.4)		
Female	244 (62.2)	244 (100)	0 (0.0)		
Age (years)				NA†	0.215
≥52	210 (53.6)	210 (100.0)	0 (0.0)		
<52	182 (46.4)	180 (98.9)	2 (1.1)		
LS genotype‡				NA†	1.000
*MLH1/MSH2/EPCAM*	255 (65.1)	254 (99.6)	1 (0.4)		
*MSH6/PMS2*	137 (34.9)	136 (99.3)	1 (0.7)		
**Did you feel anxious while using the FIT kit?**					
**Variables**	**n**	Not/slightlyanxious*, n (%)	Fairly/veryanxious*, n (%)	**χ²**	**P value**
All participants	392 (99.7)	389 (99.2)	3 (0.8)		
Sex				NA†	0.142
Male	148 (37.8)	146 (98.6)	2 (1.4)		
Female	244 (62.2)	243 (99.6)	1 (0.4)		
Age (years)				NA†	0.099
≥52	210 (53.6)	210 (100.0)	0 (0.0)		
<52	182 (46.4)	179 (98.4)	3 (1.6)		
LS genotype‡				NA†	0.555
*MLH1/MSH2/EPCAM*	255 (65.1)	252 (98.8)	3 (1.2)		
*MSH6/PMS2*	137 (34.9)	137 (100.0)	0 (0.0)		
**How confident are you that you used the FIT kit correctly?**					
**Variables**	**n**	Very/fairlyconfident*, n (%)	Not very/not at all confident*, n (%)	**χ²**	**P value**
All participants	390 (99.2)	376 (96.4)	14 (3.6)		
Sex				1.683	0.195
Male	148 (37.9)	145 (98.0)	3 (2.0)		
Female	242 (62.1)	231 (95.5)	11 (4.5)		
Age (years)				1.921	0.166
≥52	210 (53.8)	205 (97.6)	5 (2.4)		
<52	180 (46.2)	171 (95.0)	9 (5.0)		
LS genotype‡				NA†	0.778
*MLH1/MSH2/EPCAM*	254 (65.1)	244 (96.1)	10 (3.9)		
*MSH6/PMS2*	136 (34.9)	132 (97.1)	4 (2.9)		
**How confident are you that the result you will receive for the FIT kit will be accurate in detecting any potential precancer or cancer?**					
**Variables**	**n**	Very/fairlyconfident*, n (%)	Not very/not at all confident*, n (%)	**χ²**	**P value**
All participants	381 (96.9)	338 (88.7)	43 (11.3)		
Sex				0.062	0.803
Male	144 (37.8)	127 (88.2)	17 (11.8)		
Female	237 (62.2)	211 (89.0)	26 (10.1)		
Age (years)				3.972	**0.046**
≥52	205 (53.8)	**188** (**91.7**)	17 (8.3)		
<52	176 (46.2)	150 (85.2)	26 (14.8)		
LS genotype‡				0.972	0.324
*MLH1/MSH2/EPCAM*	249 (65.4)	218 (87.6)	31 (12.4)		
*MSH6/PMS2*	132 (34.6)	120 (91.0)	12 (9.0)		

**Boldface** indicates statistically significant values at p < 0.05.

* The first two responses and the last two responses within the 4-point Likert scale of this questionnaire were combined for dichotomisation purposes as required for 2×2 χ² analysis.

† Fisher’s Exact Test was used in this instance as the findings were not deemed suitable for interpretation by Pearson χ² (more than 20% of the expected frequencies had a value of <5).

‡ LS genotypes were combined in a 2×2 fashion for χ² analysis based on CRC risk and known pathogenicity.^21^

CRC, colorectal cancer; FIT, faecal immunochemical testing.

Older respondents were more likely to trust that FIT results would guide the timing of their colonoscopy (p=0.033). Female respondents were more likely to indicate that they felt ‘cared for’ by the NHS through the provision of the FIT kit (p=0.018) (table 4).

### Associations between user experience and demographics

A significant association was found between age and confidence in FIT’s accuracy, with older respondents (≥52 years) showing higher confidence than younger respondents (p=0.046).

### FIT attitudes

The FIT Attitudes Questionnaire results (table 5) showed that 82.4% of respondents found FIT reassuring, and 89.8% liked completing the FIT kit at home. Most respondents (85.0%) felt cared for by the NHS, and 86.8% trusted FIT results to guide timely colonoscopy. Notably, 92.6% believed that adding an annual FIT to biennial colonoscopy would enhance LS surveillance.

**Table 5:** Project 2 (FIT for Lynch Study) FIT attitudes

FIT attitudes items	Strongly disagree N (%)	Disagree N (%)	Indifferent N (%)	Agreen (%)	Strongly agreen (%)	No response/N/An (%)
I have been anxious about my routine colonoscopy being cancelled or postponed due to COVID-19	38 (5.7)	49 (7.3)	62 (9.2)	128 (18.9)	93 (13.7)	46 (6.8)
Having the option of using the FIT kit is reassuring	7 (1.0)	10 (1.5)	50 (7.4)	185 (27.4)	139 (20.6)	25 (3.7)
I like the idea of doing the kit in the comfort of my own home	7 (1.0)	4 (0.6)	25 (3.7)	147 (21.8)	206 (30.5)	27 (4.0)
The NHS makes me feel cared for by offering me this FIT kit while I wait for a colonoscopy	8 (1.2)	2 (0.3)	44 (6.5)	173 (25.7)	161 (23.9)	28 (4.2)
I trust that the result of the FIT test can be used to decide when I need a colonoscopy	7 (1.0)	6 (0.9)	35 (5.2)	180 (26.7)	161 (23.9)	27 (4.0)
Doing a FIT kit every year, as well as having a colonoscopy every 2 years, would improve my Lynch syndrome surveillance	7 (1.0)	1 (0.1)	13 (1.9)	133 (19.7)	231 (34.3)	31 (4.6)

Data are presented as counts and percentages (n (%)) unless otherwise stated.

FIT, faecal immunochemical testing; NHS, National Health Service.

## Discussion

This study offers valuable insights into the acceptability and perceptions of FIT among people with LS across two distinct surveillance contexts: the emergency clinical service (Project 1) and the prospective longitudinal study (Project 2). Both cohorts reported high confidence in their ability to use the FIT kit correctly and positive attitudes towards FIT as a supplemental surveillance tool. However, differences between the cohorts may reflect not only the timing of care delivery but also broader shifts in public awareness of FIT. During and after the COVID-19 pandemic, FIT was increasingly used in both screening and symptomatic contexts, likely enhancing familiarity with and perceptions of its utility.^25^,^27^ Project 1 participants, who faced greater healthcare disruptions during the pandemic, expressed slightly higher levels of anxiety and scepticism towards FIT. In contrast, Project 2 participants, recruited during a more stable postpandemic period, reported higher trust in FIT’s accuracy and greater confidence in its role in guiding timely colonoscopy.

Beyond healthcare disruptions and public awareness, clinician and patient perceptions of FIT may also be shaped by broader factors such as concerns over test sensitivity, false positives, and its evolving role in LS surveillance. While FIT has demonstrated utility in population screening and symptomatic contexts, its reliability in high-risk genetic cohorts continues to be evaluated. Future and ongoing studies should explore how communication strategies influence the acceptability and uptake of FIT-based surveillance in this patient population.

This study represents the first to assess attitudes toward FIT as a supplemental modality for colonoscopic surveillance in people with LS, providing novel insights into patient experiences across different care settings. A key strength lies in the inclusion of two large cohorts with comparable demographic and genotypic distributions, enhancing the generalisability of the findings. Additionally, both cohorts were recruited within the UK, ensuring consistency across geographical regions, and the response rate to the survey was high. Our findings align with prior research demonstrating high levels of acceptability for FIT in population-based CRC screening programmes, as well as in high-risk and postpolypectomy surveillance groups. Studies have shown that ease of use and non-invasiveness are key facilitators of FIT uptake, particularly in routine screening populations.^11 13^ Additionally, research in high-risk cohorts and postpolypectomy surveillance settings has reported strong patient confidence in FIT as a supplemental tool, with some individuals preferring it over colonoscopy due to its less invasive nature.^12 15 16^

However, FIT acceptance in LS populations remains less extensively studied. Our study builds on existing literature by identifying factors influencing FIT perceptions in patients with LS, including higher anxiety levels in women and greater confidence among older respondents. These findings highlight the importance of tailored patient communication to improve FIT uptake in this high-risk group. Future research should further explore how FIT acceptance varies across different healthcare systems and genetic risk profiles to optimise its role in LS surveillance.

While this study provides valuable insights into LS patient attitudes toward FIT within the UK NHS framework, its generalisability to other healthcare systems with different surveillance protocols remains uncertain. Given international variations in LS clinical management practices and healthcare infrastructure, further research is warranted to explore LS patient perspectives on FIT in diverse settings. Such studies will be essential to assess the feasibility and acceptability of FIT-based surveillance beyond the UK and inform tailored implementation strategies.

### Clinical implications

The findings suggest that FIT is an acceptable and valuable supplemental surveillance tool for people with LS, potentially reducing the burden associated with frequent colonoscopies. Implementing FIT in routine surveillance could improve patient adherence, especially during periods of healthcare disruption. However, addressing patient anxiety—particularly among women—will be crucial for increasing acceptance. The NHS should consider offering tailored communication strategies to alleviate concerns and foster trust in FIT as a reliable adjunct to colonoscopy. Furthermore, targeted efforts to engage non-respondents and non-participants could enhance understanding of barriers to participation and improve uptake of FIT-based surveillance. From a research perspective, future studies should explore the long-term impact of FIT implementation on surveillance outcomes and patient quality of life, as well as investigate strategies to address disparities in FIT uptake.

### Limitations

This study has several limitations. While it provides valuable cross-sectional insights into perceptions and attitudes towards FIT, it does not explore how these may evolve over time, especially as more efficacy data becomes available. Additionally, the survey design did not incorporate input from patient focus groups, which may have limited its ability to fully capture patient perspectives. Furthermore, thematic analysis from open-ended responses was limited to Project 1, with similar analysis planned for Project 2 on its completion. Finally, both studies were conducted exclusively within the UK, potentially limiting the generalisability of findings to people with LS in other healthcare contexts. However, the inclusion of two distinct cohorts recruited at different time points may address some of these limitations. For example, participants in Project 2, recruited in a more stable postpandemic period, expressed greater trust in FIT compared with those in Project 1, who experienced heightened healthcare disruptions during the pandemic. This comparison suggests that the timing of care delivery and broader familiarity with FIT may influence patient perceptions, a factor warranting further investigation in future studies.

## Conclusion

In conclusion, FIT appears to be a useful and well-accepted tool for people with LS, offering an additional layer of surveillance between routine colonoscopies. Future research should focus on understanding barriers to engagement among non-participants, as well as exploring evolving attitudes towards FIT as more data on its efficacy becomes available. Addressing feelings of anxiety, particularly among women, remains critical for ensuring broader acceptance of FIT. Multicountry collaborations may also provide valuable insights into how attitudes toward FIT differ across diverse cultural and healthcare settings, ultimately enhancing surveillance strategies for people with LS worldwide.

As FIT continues to be explored as a potential adjunct to colonoscopic surveillance in LS, further research is needed to evaluate its implementation across different healthcare systems. Understanding how CRC surveillance protocols, healthcare accessibility and patient preferences vary internationally will be essential for optimising FIT-based surveillance strategies and ensuring their effectiveness in diverse clinical settings.

## Supplementary material

10.1136/bmjgast-2025-001751 online supplemental figure 1

10.1136/bmjgast-2025-001751 online supplemental figure 2

10.1136/bmjgast-2025-001751 online supplemental figure 3

10.1136/bmjgast-2025-001751 online supplemental figure 4

10.1136/bmjgast-2025-001751 online supplemental figure 5

10.1136/bmjgast-2025-001751 online supplemental figure 6

## Data Availability

Data are available upon reasonable request.
