# Supplementary material for: Evaluation of user experiences, perceptions and attitudes towards faecal immunochemical testing (FIT) for risk-stratified colonoscopy in people with Lynch syndrome
Source: BMJ Open Gastroenterol. 2025 May 19;12(1):e001751. doi: 10.1136/bmjgast-2025-001751 (PMC12090851; doi:10.1136/bmjgast-2025-001751)
Supplement: online supplemental figure 2 [file bmjgast-12-1-s002.pdf]

Supplementary Figure 2: Project 1 (Emergency Clinical Service) Participant Survey

Full Name: _______________ DOB: _________

Patient Questionnaire:

"Rapid evaluation of Faecal immunochemical testing (FIT) levels in individuals with a Lynch Syndrome pathogenic variant to determine a revised threshold for colonoscopy in response to the COVID-19 pandemic"

We would like to ask you some questions about your experience with the faecal immunochemical test (FIT) self-sample kit and about your future bowel cancer surveillance preferences.

Please answer the below questions to the best of your ability. Your answers will be kept confidential and anonymous.

The first set of questions ask about your experience of using the faecal immunochemical test (FIT) kit for bowel cancer surveillance.

**How would you rate your overall experience of the FIT kit? (Please circle one)**			
Excellent	Good	Fair	Poor

**Did you find the FIT kit unpleasant? (Please circle one)**			
Not unpleasant	Mildly unpleasant	Fairly unpleasant	Very unpleasant

**Did you feel embarrassed using the FIT kit? (Please circle one)**			
Not at all embarrassed	Mildly embarrassed	Fairly embarrassed	Very embarrassed

**Did you feel anxious while using the FIT kit? (Please circle one)**			
Not at all anxious	Slightly anxious	Fairly anxious	Very anxious

**How confident are you that you used the FIT kit correctly? (Please circle one)**			
Yes, very confident	Yes, fairly confident	No, not very confident	No, not at all confident

**How confident are you that the result you will receive for the FIT kit will be accurate in detecting any potential precancer or cancer?**			
Yes, very confident	Yes, fairly confident	No, not very confident	No, not at all confident

The next set of questions ask about your feelings and attitudes towards routine surveillance throughout the COVID-19 pandemic and whether this may have affected your <u>future preferences</u> for bowel cancer surveillance.

Please indicate how much you agree with each of the statements below						
	**Strongly disagree**	**Disagree**	**Indifferent; Neither Agree nor Disagree**	**Agree**	**Strongly agree**	**N/A**
I have been anxious about my routine colonoscopy being cancelled or postponed due to COVID-19.	☐	☐	☐	☐	☐	☐
Having the option of using the FIT kit is reassuring.	☐	☐	☐	☐	☐	☐
I like the idea of doing the kit in the comfort of my own home	☐	☐	☐	☐	☐	☐
The NHS makes me feel cared for by offering me this FIT kit while I wait for a colonoscopy.	☐	☐	☐	☐	☐	☐
I trust that the result of the FIT test can be used to decide when I need a colonoscopy.	☐	☐	☐	☐	☐	☐
Doing a FIT kit every year, as well as having a colonoscopy every two years, would improve my Lynch Syndrome surveillance.	☐	☐	☐	☐	☐	☐

If there are other reasons why you feel that the FIT kit may or may not be an effective means of surveillance both during the COVID-19 pandemic and after, please provide your thoughts below:

....

....

How important it is to you to have your biennial colonoscopy?			
Not important	Somewhat important	Fairly important	Very important

Please provide any additional comments and/or feedback that you may have in the below section:

....

....

....

....

....

Thank you for taking the time to fill in this questionnaire
