# Supplementary material for: Evaluation of user experiences, perceptions and attitudes towards faecal immunochemical testing (FIT) for risk-stratified colonoscopy in people with Lynch syndrome
Source: BMJ Open Gastroenterol. 2025 May 19;12(1):e001751. doi: 10.1136/bmjgast-2025-001751 (PMC12090851; doi:10.1136/bmjgast-2025-001751)
Supplement: online supplemental figure 3 [file bmjgast-12-1-s003.pdf]

Supplementary Figure 3: Project 2 (FIT for Lynch Study) Participant Baseline Survey

Full Name: ________________ DOB: _________

Patient Questionnaire:

"Exploring the utility and acceptability of Faecal Immunochemical Testing (FIT) as a novel intervention for the improvement of Colorectal Cancer (CRC) surveillance in individuals with Lynch Syndrome."

We would like to ask you some questions about your experience with the faecal immunochemical test (FIT) self-sample kit and about your future bowel cancer surveillance preferences.

Please answer the below questions to the best of your ability. Your answers will be kept confidential and anonymous.

The first set of questions ask about your experience of using the faecal immunochemical test (FIT) kit for bowel cancer surveillance.

**How would you rate your overall experience of the FIT kit? (Please circle one)**			
Excellent	Good	Fair	Poor

**Did you find the FIT kit unpleasant? (Please circle one)**			
Not unpleasant	Mildly unpleasant	Fairly unpleasant	Very unpleasant

**Did you feel embarrassed using the FIT kit? (Please circle one)**			
Not at all embarrassed	Mildly embarrassed	Fairly embarrassed	Very embarrassed

**Did you feel anxious while using the FIT kit? (Please circle one)**			
Not at all anxious	Slightly anxious	Fairly anxious	Very anxious

**How confident are you that you used the FIT kit correctly? (Please circle one)**			
Yes, very confident	Yes, fairly confident	No, not very confident	No, not at all confident

**How confident are you that the result you will receive for the FIT kit will be accurate in detecting any potential precancer or cancer?**			
Yes, very confident	Yes, fairly confident	No, not very confident	No, not at all confident

The next set of questions ask about your feelings and attitudes towards routine surveillance throughout the COVID-19 pandemic and whether this may have affected your <u>future preferences</u> for bowel cancer surveillance.

Please indicate how much you agree with each of the statements below						
	Strongly disagree	Disagree	Indifferent; Neither Agree nor Disagree	Agree	Strongly agree	N/A
I have been anxious about my routine colonoscopy being cancelled or postponed due to COVID-19.	☐	☐	☐	☐	☐	☐
Having the option of using the FIT kit is reassuring.	☐	☐	☐	☐	☐	☐
I like the idea of doing the kit in the comfort of my own home	☐	☐	☐	☐	☐	☐
The NHS makes me feel cared for by offering me this FIT kit as a supplementary test prior to my colonoscopy.	☐	☐	☐	☐	☐	☐
I trust that the result of the FIT test may better inform my endoscopy and clinical teams.	☐	☐	☐	☐	☐	☐
Doing a FIT kit every year, as well as having a colonoscopy every two years, would improve my Lynch Syndrome surveillance.	☐	☐	☐	☐	☐	☐

The next set of questions will ask you about any medications that you may currently be taking, as well as any existing comorbidities / conditions that you would like to make us aware of.

<u>Note</u>: Please include aspirin if you are currently taking this on a routine basis.

Current Medications		
Medication Name	Dosage	Frequency (once/day, 2x/day, etc.)

Are you currently experiencing any notable GI-related symptoms?

____

____

____

Thank you for taking the time to fill in this questionnaire.
