# Supplementary material for: Evaluation of user experiences, perceptions and attitudes towards faecal immunochemical testing (FIT) for risk-stratified colonoscopy in people with Lynch syndrome
Source: BMJ Open Gastroenterol. 2025 May 19;12(1):e001751. doi: 10.1136/bmjgast-2025-001751 (PMC12090851; doi:10.1136/bmjgast-2025-001751)
Supplement: online supplemental figure 4 [file bmjgast-12-1-s004.pdf]

Supplementary Figure 4: Open-Ended Responses from Project 1: Negative Themes

Misinterpretation in the intended use of FIT in the context of the emergency clinical service

The FIT test may present blood in the stool as an indicator of bowel cancer but one of the reasons for having a colonoscopy can be to remove polyps at a precancerous stage. The FIT is not sufficient for this when we know Lynch cancers can be aggressive and grow quickly in the bowel.

-Female, Age Group: 36-45

...but the actual colonoscopy I find more reassuring. I have one every 18 months normally because of my genetic defect and all of the above.

-Female, Age Group: 66-75

If a colonoscopy cannot be done, then this is better than nothing, but I don't feel it should replace it.

-Female, Age Group: 36-45

I feel anyone with a genetic condition which increases chances of cancer should continue with routine screenings. I believe a colonoscopy would be more effective.
I understand the demand of COVID / the strain the NHS are under, but I feel my condition is being overlooked due to COVID-19. Cancer kills more people per day than COVID - we need our normal screenings!

-Female, Age Group: <25

I believe the FIT kit can only give so much information. I will need a colonoscopy as well when possible.

-Female, Age Group: 66-75

...I would NOT want to have colonoscopies every 2 years and would prefer to remain on yearly due to the aggressive nature of Lynch in my family.

-Female, Age Group: 56-65

...but I understand that colonoscopy is the best way of picking up the type of abnormalities we might have with Lynch Syndrome.

-Female, Age Group: 56-65

Misinterpretation as it relates to the efficacy of FIT

In my opinion, it's not a good enough test; not accurate for Lynch...

-Female, Age Group: 46-55

I don't think blood in faeces is a definitive indicator for bowel cancer.

-Female, Age Group: 66-75

I don't believe physical problem like polyps will show in a sample until after they become a problem.

-Male, Age Group: 26-35

The kit does not check if you have polyps.

-Male, Age Group: 56-65

…but I am worried that the stool sample may not be sufficient to identify the problem.
-Male, Age Group: 36-45

General misunderstanding / scepticism

Have never done this before so unsure if it would be an effective means of surveillance.
-Male, Age Group: 26-35

I have had polyps removed on every colonoscopy I have had. I am concerned that polyps may reach cancerous state by the time blood is detected.
-Male, Age Group: 36-45

The colonic adenomas could get quite big before they bleed and get picked up by FIT.
-Male, Age Group: 56-65

If the FIT test is only looking for blood, surely it will miss a growth. Perhaps I need more information.
-Female, Age Group: 56-65

Valid or applicable clinical concerns

I could have a small polyp or tumour that isn't bleeding and the test will not detect them.
-Male, Age Group: 56-65

I have haemorrhoids so concerned that FIT kit might have false positive?
-Male, Age Group: 36-45

My father's bowel cancer was not detected using this method.
-Female, Age Group: 26-35

Not sure, as I occasionally have slight bleeding from small haemorrhoids or soreness after irritable bowel.
-Female, Age Group: 66-75

The bowel screening programme did not show up anything prior to my bowel cancer being diagnosed. This tends to make me think that a colonoscopy will pick up more bowel cancers earlier.
-Male, Age Group: 66-75

This form doesn't ask any questions about symptoms. Like I had blood in my passage and the whole toilet for a whole day…
-Female, Age Group: 46-55

Concern regarding the design or manufacturing of the FIT device

I'm not sure the FIT kit grooves are suitable for soft stools. However, it is my first time using it.
-Male, Age Group: 26-35

Reported concern or anxiety as it relates to the lack of transparent FIT results as part of this emergency clinical service

Not having the results made me feel extremely anxious (e.g., could have gotten lost in the post). It was not obvious how to get the results (small print on leaflet)

-Female, Age Group: 46-55

I am obviously anxious with the procedure and the possible outcome…

-Female, Age Group: 66-75
