# Supplementary material for: Evaluation of user experiences, perceptions and attitudes towards faecal immunochemical testing (FIT) for risk-stratified colonoscopy in people with Lynch syndrome
Source: BMJ Open Gastroenterol. 2025 May 19;12(1):e001751. doi: 10.1136/bmjgast-2025-001751 (PMC12090851; doi:10.1136/bmjgast-2025-001751)
Supplement: online supplemental figure 5 [file bmjgast-12-1-s005.pdf]

Supplementary Figure 5: Open-Ended Responses from Project 1: Neutral Themes

Lack of opinion due to lack of information or education on the topic

… However, like any medical procedure, I don't know the difference in results from a FIT to a full colonoscopy.

-Male, Age Group: 36-45

Cautious optimism with clarification requested around perceived outcome

I think a good idea to use in conjunction with colonoscopies but not instead of.

-Female, Age Group: 46-55

I believe the use of the FIT kit would be good in addition to, but not instead of my routine colonoscopy.

-Male, Age Group: 26-35

…it would be good to have information to show whether the FIT test works with adequate sensitivity and specificity in Lynch patients to pick up colonic adenoma/cancer.

-Male, Age Group: 56-65

Pleased that any intervention was offered during this time at all (in lieu of regular surveillance colonoscopy) with no clear opinion on FIT itself

…better than nothing so merci!

-Female, Age Group: 46-55

I'm not sure being [illegible] how effective the FIT test is but feel that anything is better than nothing at all.

-Female, Age Group: 56-65
