# Supplementary material for: Evaluation of user experiences, perceptions and attitudes towards faecal immunochemical testing (FIT) for risk-stratified colonoscopy in people with Lynch syndrome
Source: BMJ Open Gastroenterol. 2025 May 19;12(1):e001751. doi: 10.1136/bmjgast-2025-001751 (PMC12090851; doi:10.1136/bmjgast-2025-001751)
Supplement: online supplemental figure 6 [file bmjgast-12-1-s006.pdf]

Supplementary Figure 6: Open-Ended Responses from Project 1: Positive Themes

Welcomed the idea of routinely utilising FIT as part of ongoing CRC surveillance

I think it will be good to do one every year...

-Male, Age Group: 66-75

Because of my family history of bowel cancer, and my cancer of the uterus, I imagine that this test is worthwhile.

-Female, Age Group: 66-75

Having the FIT kit is better than having no surveillance. I would feel confident doing this test more often.

-Female, Age Group: 56-65

Enthusiastic about FIT and confident in ability to yield accurate results

I think it's a great idea to have it and I trust they will give results...

-Male, Age Group: 26-35

Expressed reassurance in having a supplementary modality to colonoscopy

It's an extra option to make sure all is well.

-Female, Age Group: 66-75

This along with my colonoscopy would be reassuring.

-Female, Age Group: 56-65

General gratitude towards NHS for the emergency clinical service and in considering the welfare of the LS patient population despite endoscopic limitations in response to the COVID-19 pandemic

I am very impressed with endoscopy's concern for my welfare considering the pressures imposed by the pandemic

-Male, Age Group:56-65

Reassuring to know that I haven't been overlooked for my 2 yearly colonoscopy and that all that is possible is being done in these uncertain times. Thank you.

-Female, Age Group: 66-75

I was pleased to receive the FIT kit as an intermediary survey and it certainly could expose cancer. Thank you for considering Lynch Syndrome patients in this way. Thank you also for all the work in trying to keep the screening programmes going at such a difficult time.

-Female, Age Group: 36-45

I think it's an excellent idea. Saves time traveling to Manchester. It's reassuring that you're offering these tests in a time when the NHS is fighting a pandemic you're still looking after your patients. Thank you.

-Female, Age Group: 46-55

Surprised that they do this but happy that I am still on the system to be checked and looked after during this pandemic as I did feel that I would be overlooked. Thanks.

-Male, Age Group: 56-65

Thanks for your support it's invaluable. Merci!

-Female, Age Group: 46-55

I am very grateful for the care I get from the team at the Harrow.

-Female, Age Group: 66-75

I am always impressed by the care I have received in the pursuit of treatment for my Lynch

-Male, Age Group: 36-45

I am reassured that this system demonstrates that the NHS is monitoring my condition

-Male, Age Group: 36-45

...grateful that the NHS can offer this service.

-Female, Age Group: 66-75

My 46-year-old maternal cousin who is also celiac and has Lynch has just had a Whipple procedure to remove a tumour in her pancreas. It's imperative I have these preventative measures done. Thank you.

-Female, Age Group: 46-55

I have been under the [illegible] team for 30 years as my father and his brother all died of bowel cancer. There was not the screening then like there is today. If I had not been screened, I might not have been here...

-Female, Age Group: 66-75

Partial to annual FIT with less frequent colonoscopies

I feel happier doing the FIT kit and would like to continue doing one annually w/ maybe a bigger gap between colonoscopies. Thank you.

-Male, Age Group: 26-35

Favourable towards FIT in general

Think the FIT kit is an excellent idea...

-Female, Age Group: 46-55

It is good to have the FIT test…

-Female, Age Group: 56-65

Personally, think this is an amazing idea and will hopefully bring reassurance to many people.

-Male, Age Group: 36-45

Expressed preference of FIT over colonoscopy

I hate having a colonoscopy because I cannot stand drinking the laxative. Any alternative is very welcome.

-Female, Age Group: 66-75
